# Structural Magnetic Resonance Imaging Demonstrates Abnormal Regionally-Differential Cortical Thickness Variability in Autism: From Newborns to Adults

**DOI:** 10.3389/fnhum.2019.00075

**Published:** 2019-03-14

**Authors:** Jacob Levman, Patrick MacDonald, Sean Rowley, Natalie Stewart, Ashley Lim, Bryan Ewenson, Albert Galaburda, Emi Takahashi

**Affiliations:** ^1^Division of Newborn Medicine, Department of Medicine, Boston Children's Hospital, Harvard Medical School Boston, MA, United States; ^2^Athinoula A. Martinos Center for Biomedical Imaging, Massachusetts General Hospital, Harvard Medical School Charlestown, MA, United States; ^3^Department of Mathematics, Statistics and Computer Science, St. Francis Xavier University Antigonish, NS, Canada; ^4^Department of Neurology, Beth Israel Deaconess Medical Center, Harvard Medical School Boston, MA, United States

**Keywords:** autistic, cortical thickness, development, neuroanatomy, variability

## Abstract

Autism is a group of complex neurodevelopmental disorders characterized by impaired social interaction and restricted/repetitive behavior. We performed a large-scale retrospective analysis of 1,996 clinical neurological structural magnetic resonance imaging (MRI) examinations of 781 autistic and 988 control subjects (aged 0–32 years), and extracted regionally distributed cortical thickness measurements, including average measurements as well as standard deviations which supports the assessment of intra-regional cortical thickness variability. The youngest autistic participants (<2.5 years) were diagnosed after imaging and were identified retrospectively. The largest effect sizes and the most common findings not previously published in the scientific literature involve abnormal intra-regional variability in cortical thickness affecting many (but not all) regions of the autistic brain, suggesting irregular gray matter development in autism that can be detected with MRI. Atypical developmental patterns have been detected as early as 0 years old in individuals who would later be diagnosed with autism.

## Introduction

Autism is characterized by impaired social communication, deficits in social reciprocity and repetitive/stereotyped behaviors (Gillberg, 1993; Wing, 1997). Evidence for the existence of neuroanatomical differences between participants with autism and control subjects comes from a variety of postmortem and neuroimaging research (Toal et al., 2005; Amaral et al., 2008). Magnetic resonance imaging (MRI) provides a wide variety of physiological/anatomical measurements of a participant's brain, information that may assist in both clinical applications and basic research. The most commonly used MRI method produces structural information related to the concentration of hydrogen protons, providing clinically useful soft tissue contrast. In the brain, structural MRI provides for the ability to differentiate between gray matter, white matter and cerebrospinal fluid, which forms the basis for the extraction of a variety of measurements distributed across brain regions, such as white matter volume measurements, cortical thickness measurements, cortical folding/gyration-based measurements, cortical surface area measurements, and more (Fischl, 2012).

**Figure 1:**
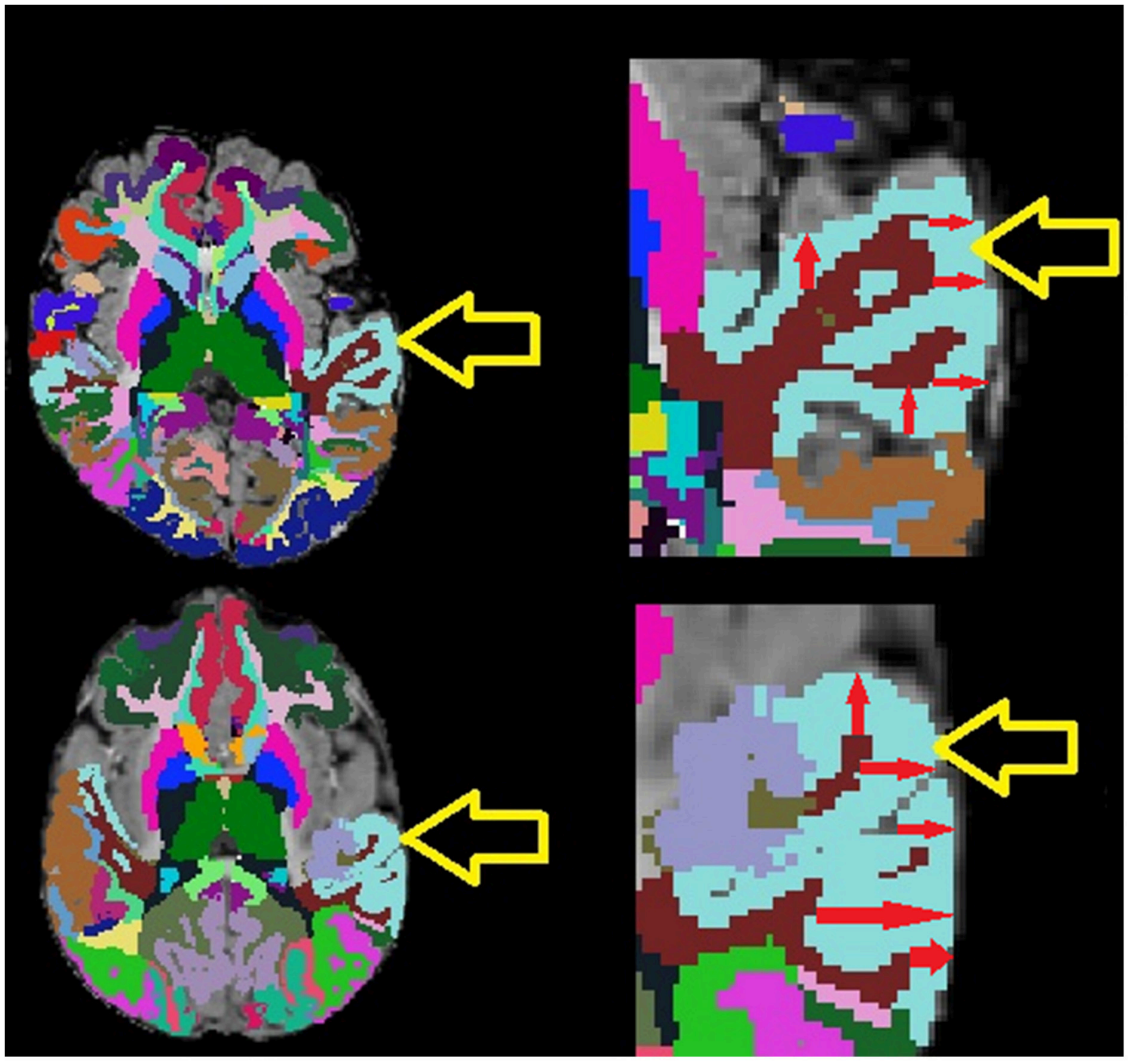
Example MRI examinations with FreeSurfer ROIs (colored regions) with magnifications. Yellow arrows point to the light blue superior temporal cortex, red arrows provide demonstrative examples of some of the many within-region cortical thickness measurements computed by FreeSurfer. Note that the exam on top exhibits lower cortical thickness variability than the exam on the bottom which exhibits highly varying cortical thickness measurements.

The analysis of autistic participants who have undergone structural MRI examinations has been the subject of many studies in the literature that have incorporated distributed quantification of volumes, cortical thicknesses, surface areas etc. with automated biomarker extraction technologies, such as FreeSurfer (Fischl, 2012). However, existing studies have been limited in the populations assessed, providing incomplete data regarding the developmental stages of autistic participants, particularly in terms of the ages of participants included in the analysis and the number of participants included in the age range being evaluated (Dziobek et al., 2010; Ecker et al., 2010, 2013, 2014; Groen et al., 2010; Jiao et al., 2010; Schumann et al., 2010; Schaer et al., 2013, 2015; Wallace et al., 2013; Zielinski et al., 2014; Lefebvre et al., 2015; Richter et al., 2015; Haar et al., 2016; Yang et al., 2016). Furthermore, although investigating average cortical thickness is common in the literature (Jiao et al., 2010; Ecker et al., 2013, 2014; Zielinski et al., 2014; Yang et al., 2016), none of the studies appears to have considered intra-regional cortical thickness variability as a measurement of potential interest in autism. Intra-regional cortical thickness variability measurements are readily available in FreeSurfer (Fischl, 2012) in the form of the standard deviation of within-region cortical thickness measurements. Examples of two examinations exhibiting differing cortical thickness standard deviation measurements are provided in Figure 1 to illustrate intra-regional cortical thickness variability to the reader.

It is particularly challenging to assess imaging features of autism in a pediatric population, because of the structural changes between children and adults (Reiss et al., 1996; Casey et al., 1997; Thomas et al., 2001; Bunge et al., 2002; Gogtay et al., 2004; Fair et al., 2009; Supekar et al., 2009). Important information regarding brain function is encoded in distributed patterns of brain activity and structure (Mesulam, 1981; Vaadia et al., 1995; McIntosh et al., 1996; Fox et al., 2005), and identifying these patterns is particularly challenging in a pre-adult population, because of a rapidly changing anatomy and physiology, a high degree of brain plasticity, small brain sizes, participant motion, and an incomplete understanding of brain development.

In the present study, we hypothesize that the assessment of cortical thickness from clinical structural MRI examinations has the potential to assist in the diagnosis of autism and to improve our understanding of brain physiology associated with the condition. This study attempts to provide a thorough assessment of the clinical potential for structural MRI in assessing cortical thickness by including all available autistic participants who received MRI examinations at Boston Children's Hospital (BCH) at 3 Tesla producing volumetric T1 examinations compatible with the automated extraction of distributed measurements (Fischl, 2012). We hypothesize that regional differences in average cortical thickness and cortical thickness variability measurements are associated with the clinical presentation of the autistic brain and can be identified by structural MRI. These differences were assessed individually in each identified brain region in order to see whether our analysis is sensitive to region-specific neurodevelopmental abnormalities associated with autism.

## Materials and Methods

### Participants

**Figure 2:**
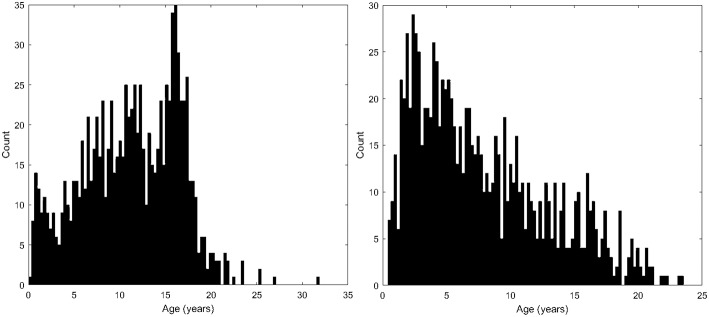
Histograms demonstrating the population age distributions in this study's neurotypical participants **(left)** and autistic participants **(right)**.

Following approval by BCH's Institutional Review Board (informed consent was waived due to the lack of risk to participants included in this retrospective analysis), the clinical imaging electronic database at BCH was reviewed for the present analysis from 01/01/2008 until 02/24/2016, and all brain MRI examinations of participants aged 0 to 32 years at the time of imaging were included for further analysis if autism was indicated in the participant's electronic medical records. More detailed diagnostic information (such as Autism Diagnostic Interview, Revised–ADI-R and Autism Diagnostic Observation Schedule–ADOS gold standard diagnoses) were not available in this dataset and this issue is addressed in more detail in the limitations section of the discussion. Examinations deemed to be of low quality (because of excessive participant motion, large metal artifact from a participant's dental hardware, lack of a T1 structural imaging volume providing diagnostically useful axial, sagittal and coronal oriented images etc.) were excluded from the study. Examinations that were inaccessible for technical reasons were also excluded. This yielded 1,003 examinations from 781 autistic participants. Control subjects were assembled retrospectively in a previous analysis (Levman et al., 2017) by selecting participants on the basis of a normal MRI examination, as assessed by a BCH neuroradiologist, and whose medical records provided no indication of any neurological problems (participants with any known disorder were excluded such as autism, cerebral palsy, traumatic brain injury, brain cancer, developmental delay, multiple sclerosis, tuberous sclerosis complex, stroke, neurofibromatosis, cortical dysplasia, epilepsy, attention deficit hyperactivity disorder, etc.). Participants with any form of non-neurological cancer were also excluded to avoid data exhibiting growth trajectories negatively affected by treatments such as chemotherapy. The same exclusion criteria applied to the autistic population were also applied to the control subjects. This yielded 993 examinations from 988 control subjects. Histograms demonstrating the age distributions for both the control subjects and autistic groups are provided in Figure 2. Table 1 provides a breakdown of the autistic and healthy populations divided by age groups used in the statistical analysis section of this manuscript's Methods.

**Table 1:** Demographic breakdown of our two populations.

**Group**	**0 to 5 years**	**5 to 10 years**	**10 to 15 years**	**15 to 20 years**	**20+ years**
Neurotypical	M = 71, F = 68	M = 124, F = 137	M = 115, F = 177	M = 80, F = 194	M = 4, F = 23
	2.59 ± 1.43 yrs	7.63 ± 1.41 yrs	12.41 ± 1.41 yrs	16.70 ± 1.11 yrs	22.21 ± 2.63 yrs
Autistic	M = 278, F = 101	M = 242, F = 74	M = 144, F = 37	M = 97, F = 14	M = 12, F = 4
	2.89 ± 1.19 yrs	7.23 ± 1.47 yrs	12.17 ± 1.43 yrs	16.97 ± 1.42 yrs	21.27 ± 1.01 yrs

*M, count of males; F, count of females; each are followed by the average and standard deviation of this group's ages in years (yrs)*.

### MRI Data Acquisition and Preprocessing

Participants were imaged with clinical 3 Tesla MRI scanners (Skyra, Siemens Medical Systems, Erlangen, Germany) at BCH yielding T1 structural volumetric images accessed through the Children's Research and Integration System (Pienaar et al., 2014). Because of the clinical and retrospective nature of this study, there is variability in the pulse sequences employed to acquire these volumetric T1 examinations. Spatial resolution varied in the x and y directions from 0.219 to 1.354 mm (mean: 0.917 mm, standard deviation: 0.124 mm). Through-plane slice thickness varied from 0.500 to 2.000 mm (mean: 0.996 mm, standard deviation: 0.197 mm). Strengths and limitations of the large-scale varying MR protocol approach taken in this study are addressed in the Discussion. Motion correction was not performed, but examinations with substantial motion artifacts were carefully excluded based on visual assessment. These motion corruption exclusions were performed to compensate for the additional difficulties autistic patients have remaining still during image acquisition relative to the control subjects. T1 structural examinations were processed with FreeSurfer (Fischl, 2012) using the recon_all command which aligns the input examination to all available atlases. Those atlases that include cortical thickness measurements were included for further analysis (aparc, aparc.a2009, aparc.DKTatlas40, BA, BA.thresh, entorhinal_*exvivo*). These combined atlases include definitions of 331 cortical regions. Each FreeSurfer output T1 structural examination was displayed with label map overlays and visually inspected for quality of regional segmentation results. If FreeSurfer results were observed to substantially fail, they were excluded from this analysis (i.e. FreeSurfer regions-of-interest (ROIs) that do not align to the MRI and examinations where major problems were observed with an ROI such as a cerebellar segmentation extending far beyond the extent of the cerebellum).

### Statistical Analysis

This study included the acquisition of 662 regionally distributed cortical thickness measurements per imaging examination, as extracted by FreeSurfer's recon-all command which processes the input examination with all available atlases (Fischl, 2012). This included extracting measurements of both average and the standard deviation of within-region cortical thicknesses for each supported gray matter region. This includes all sub-regions of the brain supporting cortical thickness measurements across all FreeSurfer supported atlases. Study participants were divided into four groups based on age: early childhood (0–5 years old), late childhood (5–10 years old), early adolescence (10–15 years old), and late adolescence (15–20 years old). We had very few participants older than 20 years and so did not include them in a separate group, however, all scatter plots included all participants regardless of age to facilitate visual comparison. Trend lines in all scatter plots were established with a rolling average (K = 150) implemented in MATLAB. We are interested in the diagnostic potential of these clinically acquired measurements and so each measurement (as extracted by FreeSurfer) within each age range was compared in a group-wise manner (autism compared with control subjects) with receiver operating characteristic (ROC) curve analysis which is summarized with the area under the ROC curve (AUC) (Youngstrom, 2014), Cohen's d statistic (positive/negative values indicate a higher/lower average value in the autistic population relative to the control subjects) and a *p*-value based on the standard *t*-test (Student, 1908) for two groups of samples. The *p*-value was selected as an established method to demonstrate that it is unlikely that our findings were the result of random chance, Cohen's d was selected as it is the most established method to assess effect sizes and the AUC was selected to extend our analysis to the assessment of diagnostic potential. This yielded a total of m = 2,648 group-wise comparisons, yielding a Bonferroni corrected threshold for achieving statistical significance of *p* < 0.05/m = 1.89e^−5^.

In order to confirm that the findings reported are the result of group-wise differences between the autistic and control subjects, a statistical model was constructed based on multivariate regression using MATLAB's (Natick, MA) mvregress function, adjusting each measurement within each age range in order to control for group-wise differences in age, gender, estimated total intracranial volume and the leading comorbid status of the most common secondary conditions from our two groups: headaches (7% in the autistic group, 19% in the control subjects), attention deficit hyperactivity disorder / ADHD (16% in the autistic group, 0% in the control subjects), epilepsy (13% in the autistic group, 0% in the control subjects), global developmental delay (26% in the autistic group, 0% in the control subjects), migraines (3% in the autistic group, 23% in the control subjects), and abdominal pain (14% in the autistic group, 11% in the control subjects). This model was used to adjust each cortical thickness (mean and standard deviation) measurement, in order to evaluate whether group-wise differences between our autistic and control subjects are the result of age, gender, intracranial volume or comorbid effects.

A preliminary statistical validation was performed on the independently acquired Autism Brain Imaging Data Exchange (ABIDE) dataset (Di Martino et al., 2014). The ABIDE dataset is a multi-center study with variability in data acquisition between centers. We have elected to perform a preliminary validation analysis assessing the leading five feature measurements identified in our findings (first five rows of Table 2) against the single ABIDE imaging center with the most participants aged 15–20 at imaging (the USM-ABIDE data) as this age range exhibited the largest group-wise differences in our study. Raw (unadjusted) measurements extracted by FreeSurfer from the ABIDE dataset are directly compared with raw (unadjusted) measurements extracted by FreeSurfer from our large clinical BCH dataset.

**Table 2:** Age-dependent ROC Analysis Results – Leading Measurements by AUC with Cohen's d statistic.

**Cortical measurement of interest**	**Measurement type (CT)**	**Ages 0–5 years L&R: AUC/d**	**Ages 5–10 years L&R: AUC/d**	**Ages 10–15 years L&R: AUC/d**	**Ages 15–20 years L&R: AUC/d**
Superior temporal cortex	Variability	L (0.58/−0.29) R (0.59/−0.34)	L (0.59/0.33) R (0.64/0.45)	L (0.64/0.54) R (0.63/0.49)	L (0.73/0.83) R (0.75/0.91)
Middle occipital gyrus	Variability	L (0.62/−0.40) R (0.60/−0.35)	L (0.65/0.54) R (0.62/0.44)	L (0.63/0.50) R (0.62/0.42)	L (0.74/0.90) R (0.71/0.72)
Superior parietal cortex	Variability	L (0.67/−0.57) R (0.67/−0.51)	L (0.53/0.12) R (0.54/0.16)	L (0.60/0.42) R (0.59/0.39)	L (0.71/0.74) R (0.74/0.90)
Brodmann's area 6	Variability	L (0.62/−0.37) R (0.64/−0.40)	L (0.56/0.19) R (0.54/0.12)	L (0.63/0.44) R (0.61/0.38)	L (0.73/0.78) R (0.71/0.70)
Superior temporal sulcus	Variability	L (0.66/−0.52) R (0.65/−0.49)	L (0.63/0.47) R (0.62/0.43)	L (0.67/0.63) R (0.62/0.43)	L (0.73/0.85) R (0.68/0.76)
Brodmann's area 18 (V2)	Variability	L (0.67/−0.61) R (0.62/−0.37)	L (0.60/0.45) R (0.55/0.26)	L (0.70/0.73) R (0.60/0.45)	L (0.73/0.91) R (0.67/0.71)
middle temporal visual area	Variability	L (0.63/−0.43) R (0.62/−0.50)	L (0.64/0.53) R (0.67/0.55)	L (0.67/0.60) R (0.66/0.57)	L (0.72/0.83) R (0.72/0.73)
Inferior temporal cortex	Variability	L (0.63/−0.41) R (0.61/−0.37)	L (0.60/0.42) R (0.60/0.35)	L (0.62/0.49) R (0.59/0.31)	L (0.72/0.85) R (0.70/0.72)
Brodmann's area 1	Variability	L (0.61/−0.35) R (0.61/−0.35)	L (0.52/0.00) R (0.50/−0.01)	L (0.55/0.20) R (0.55/0.14)	L (0.66/0.57) R (0.72/0.76)
Triangular part of inferior frontal gyrus	Variability	L (0.60/−0.35) R (0.59/−0.31)	L (0.57/0.17) R (0.57/0.17)	L (0.56/0.19) R (0.57/0.19)	L (0.69/0.68) R (0.72/0.78)
Supramarginal gyrus	Variability	L (0.62/−0.41) R (0.65/−0.53)	L (0.51/−0.06) R (0.53/0.07)	L (0.61/0.32) R (0.59/0.24)	L (0.72/0.73) R (0.67/0.53)
Superior parietal lobule	Variability	L (0.66/−0.53) R (0.66/−0.49)	L (0.52/0.06) R (0.52/0.09)	L (0.58/0.28) R (0.57/0.27)	L (0.67/0.59) R (0.71/0.79)
Banks of the superior temporal sulcus	Variability	L (0.60/−0.34) R (0.60/−0.26)	L (0.58/0.28) R (0.59/0.30)	L (0.61/0.48) R (0.59/0.36)	L (0.71/0.78) R (0.71/0.73)
Intraparietal sulcus	Variability	L (0.65/−0.54) R (0.67/−0.51)	L (0.50/0.03) R (0.54/0.15)	L (0.63/0.44) R (0.61/0.39)	L (0.70/0.64) R (0.71/0.77)
Brodmann's area 18 (V2)	Average	L (0.59/−0.25) R (0.59/−0.24)	L (0.59/0.33) R (0.58/0.32)	L (0.71/0.77) R (0.67/0.67)	L (0.67/0.71) R (0.71/0.82)
Inferior parietal cortex	Variability	L (0.66/−0.47) R (0.65/−0.50)	L (0.59/0.37) R (0.60/0.34)	L (0.64/0.51) R (0.61/0.43)	L (0.71/0.78) R (0.70/0.71)
Pars triangularis	Variability	L (0.61/−0.36) R (0.61/−0.33)	L (0.53/0.09) R (0.53/0.11)	L (0.52/0.08) R (0.58/0.26)	L (0.67/0.58) R (0.71/0.76)
Precuneus cortex	Variability	L (0.67/−0.57) R (0.67/−0.56)	L (0.54/0.11) R (0.56/0.17)	L (0.58/0.29) R (0.63/0.47)	L (0.69/0.69) R (0.71/0.72)
Medial orbitofrontal cortex	Average	L (0.63/−0.31) R (0.63/−0.30)	L (0.64/0.51) R (0.61/0.48)	L (0.71/0.78) R (0.67/0.69)	L (0.67/0.68) R (0.61/0.53)
Brodmann's area 2	Variability	L (0.64/−0.48) R (0.63/−0.42)	L (0.53/0.13) R (0.53/0.10)	L (0.54/0.21) R (0.58/0.29)	L (0.64/0.51) R (0.71/0.67)
Middle temporal gyrus	Variability	L (0.55/−0.20) R (0.61/−0.32)	L (0.56/−0.26) R (0.58/0.33)	L (0.59/0.35) R (0.65/0.56)	L (0.70/0.69) R (0.70/0.74)
Brodmann's area 45	Variability	L (0.61/−0.36) R (0.57/−0.26)	L (0.56/0.18) R (0.57/0.21)	L (0.54/0.14) R (0.59/0.23)	L (0.66/0.55) R (0.70/0.71)
Superior frontal cortex	Variability	L (0.63/−0.43) R (0.63/−0.44)	L (0.52/0.03) R (0.50/0.01)	L (0.59/0.32) R (0.60/0.33)	L (0.70/0.69) R (0.67/0.65)
Gyrus rectus	Average	L (0.61/−0.28) R (0.59/−0.21)	L (0.64/0.50) R (0.61/0.44)	L (0.70/0.75) R (0.61/0.48)	L (0.64/0.57) R (0.53/0.18)
Lateral orbitofrontal cortex	Average	L (0.62/−0.23) R (0.58/−0.01)	L (0.60/0.33) R (0.57/0.29)	L (0.67/0.60) R (0.70/0.71)	L (0.65/0.56) R (0.63/0.52)
Lateral aspect of superior temporal gyrus	Variability	L (0.59-0.26) R (0.59/−0.29)	L (0.50/0.05) R (0.57/0.23)	L (0.58/0.33) R (0.58/0.37)	L (0.70/0.74) R (0.70/0.69)
Middle frontal gyrus	Variability	L (0.66/−0.53) R (0.63/−0.48)	L (0.52/−0.04) R (0.51/−0.06)	L (0.54/0.17) R (0.55/0.18)	L (0.69/0.64) R (0.70/0.69)
Precentral cortex	Variability	L (0.60/−0.34) R (0.60/−0.30)	L (0.55/0.19) R (0.54/0.17)	L (0.57/0.27) R (0.53/0.11)	L (0.70/0.73) R (0.66/0.57)
Medial orbital sulcus	Average	L (0.60/−0.28) R (0.56/−0.16)	L (056/0.32) R (0.58/0.35)	L (0.63/0.53) R (0.68/0.65)	L (0.64/0.56) R (0.70/0.75)
Fusiform cortex	Variability	L (0.60/−0.35) R (0.59/−0.25)	L (0.69/0.69) R (0.65/0.52)	L (0.66/0.63) R (0.63/0.49)	L (0.65/0.62) R (0.66/0.64)
Occipital pole	Average	L (0.51/0.09) R (0.51/0.04)	L (0.59/0.33) R (0.62/0.44)	L (0.69/0.68) R (0.67/0.65)	L (0.67/0.61) R (0.69/0.74)
Lateral occipital Cortex	Variability	L (0.65/−0.48) R (0.63-0.42)	L (0.60/0.42) R (0.55/0.27)	L (0.59/0.38) R (0.61/0.38)	L (0.69/0.78) R (0.66/0.55)
Caudal middle frontal cortex	Variability	L (0.64/−0.44) R (0.62/−0.39)	L (0.52/−0.03) R (0.50/−0.02)	L (0.55/0.18) R (0.56/0.22)	L (0.69/0.67) R (0.69/0.63)
Paracentral sulcus and lobule	Variability	L (0.64/−0.44) R (0.60/−0.30)	L (0.51/0.05) R (0.52/0.10)	L (0.55/0.17) R (0.53/0.18)	L (0.63/0.41) R (0.69/0.66)
Brodmann's area 17 (V1)	Average	L (0.62/−0.37) R (0.61/−0.29)	L (0.64/0.50) R (0.57/0.30)	L (0.69/0.71) R (0.65/0.61)	L (0.65/0.60) R (0.62/0.48)
Lingual cortex	Variability	L (0.64/−0.53) R (0.64/−0.40)	L (0.53/0.19) R (0.51/0.12)	L (0.55/0.26) R (0.52/0.13)	L (0.69/0.71) R (0.58/0.32)
Lateral occipital cortex	Average	L (0.51/0.12) R (0.49/0.01)	L (0.50/0.01) R (0.54/0.17)	L (0.63/0.44) R (0.65/0.56)	L (0.69/0.69) R (0.69/0.70)
H-shaped orbital sulcus	Variability	L (0.51/−0.08) R (0.55/−0.11)	L (0.51/0.04) R (0.51/0.02)	L (0.53/0.04) R (0.57/0.27)	L (0.59/0.31) R (0.69/0.67)
Isthmus cingulate cortex	Variability	L (0.54/−0.12) R (0.54/−0.11)	L (0.56/0.23) R (0.58/0.35)	L (0.65/0.52) R (0.69/0.63)	L (0.63/0.52) R (0.61/0.40)
Cuneus gyrus	Average	L (0.56/−0.21) R (0.58/−0.24)	L (0.63/0.49) R (0.59/0.36)	L (0.69/0.75) R (0.65/0.60)	L (0.64/0.62) R (0.61/0.54)
Inferior part of the precentral sulcus	Variability	L (0.62/−0.43) R (0.64/−0.51)	L (0.53/0.10) R (0.50/−0.01)	L (0.57/0.21) R (0.59/0.28)	L (0.69/0.62) R (0.68/0.55)

*R, right; L, left; CT, cortical thickness; Variability, standard deviation of cortical thickness; AUC, area under the ROC curve; d, Cohen's d statistic*.

## Results

Many brain regions showed Bonferroni-corrected, statistically significant differences in cortical thickness measurements between participants with autism and control subjects (Table 2). Namely, there were a large number of regions of the brain exhibiting abnormal intra-regional variability in cortical thickness as measured with the standard deviation. Average cortical thickness differences between our control subjects and autistic participants were also observed across several regions of the brain (Table 2). Of the 2,648 group-wise comparisons performed, 21.9% exceeded the Bonferroni correction for statistical significance, indicating that many brain regions did not exhibit abnormal presentation of cortical thicknesses. Each measurement in Table 2 exceeds the Bonferroni correction in at least one age group, however, all age groupings are provided for ease of comparison.

The age-dependent, d statistic and ROC curve analyses yielded a variety of measurements that offer diagnostic potential and may help elucidate the underlying anatomical and physiological conditions associated with autism. Table 2 presents the leading measurements organized by AUC (highest AUC values are found at the top of the table), along with the associated d statistic as computed from the unadjusted measurement data produced by FreeSurfer. Thus, the superior temporal gyrus exhibits the most separation between groups (ages 15–20), the second most separation is found in the middle occipital gyrus (ages 15–20), etc. Our statistically adjusted data using multivariate-regression exhibits decreased *p*-values and increased separation between our autistic and control subjects relative to the raw data extracted with FreeSurfer. This was performed to confirm that the findings reported are not the result of age, gender, intracranial volume, or comorbid effects. We elected to present the raw results rather than the adjusted results because of the potential role of raw data in future diagnostic technologies and for ease of comparison with future studies.

**Figure 3:**
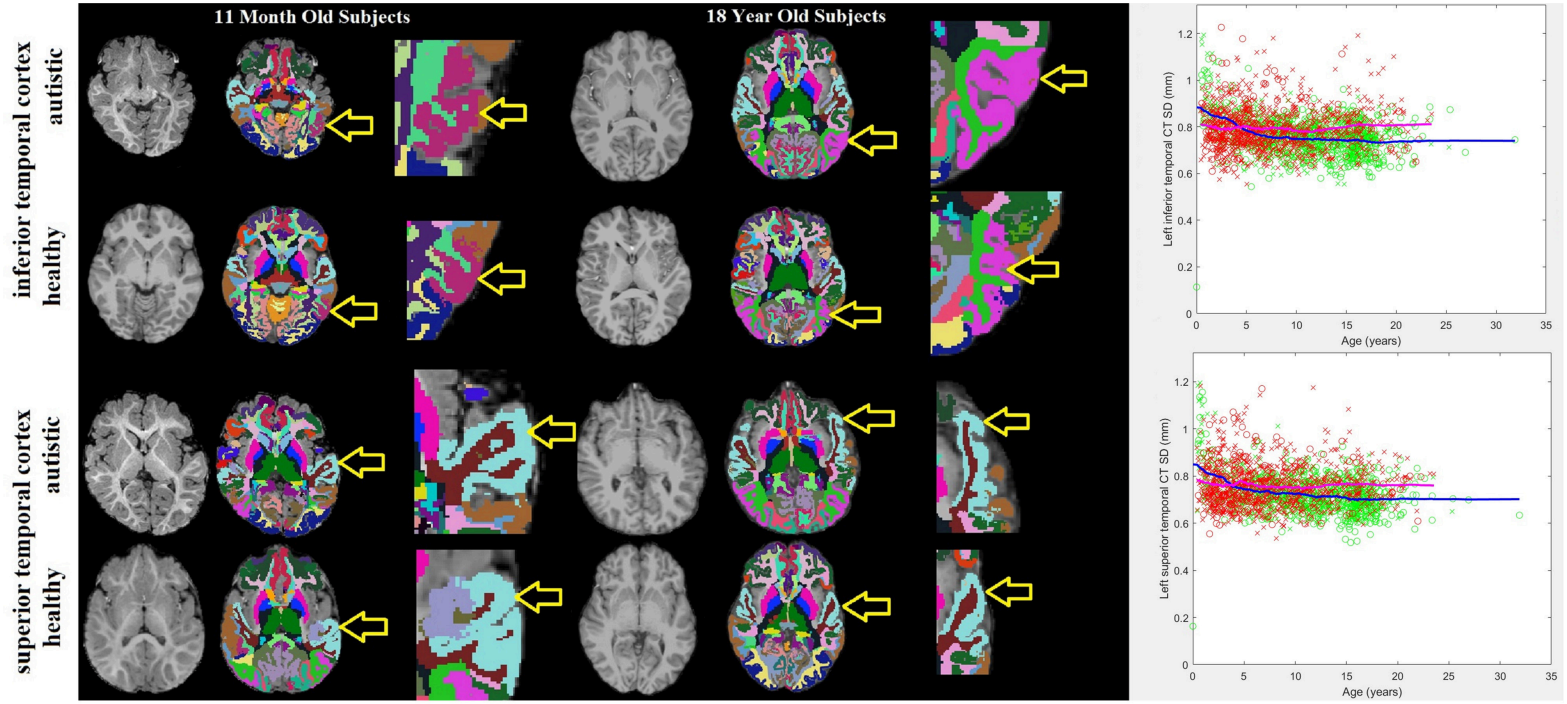
11-months-old and 18-years-old participants with autism and typically developing controls. Three images are provided for each participant: the MRI examination **(left)**, the automatically generated ROI with colors representing sub-regions of the brain identified **(center)** and a magnified image of the tissue-of-interest's ROI **(right)**. Yellow arrows point to the purple shaded inferior temporal cortex ROI and the light blue shaded superior temporal cortex ROI. To the far right are scatter plots of age variations with the standard deviation of the thickness of the left inferior and superior temporal cortices in autistic participants (red) and neurotypical controls (green). Male participants are represented by an “x,” females with an “o.” Trend lines are plotted (magenta = autistic, blue = neurotypical).

Histograms demonstrating the age distributions for both the control subjects and autistic groups are provided in Figure 2. Examples of distributed gray and white matter ROIs of longitudinal relaxation (T1) structural MRI examinations of 11-months-old and 18-years-old autistic and control subjects are shown in Figure 3 (left panel). The variability (standard deviation) of the thickness of the inferior and superior temporal cortices (yellow arrows in Figure 3) demonstrate group-wise differences between our autistic and control groups. Magnified ROIs are provided to assist in visualization of differences in cortical thickness variability. Note the reduced cortical thickness variability in the young autistic participant (bottom) as compared with the control subject (top) and the inversion of this effect among older participants. Scatter plots of the standard deviation (*SD*) of the cortical thickness in both our autistic and control subjects (Figure 3, right side) demonstrate age variability and gender.

Table 2 demonstrates that the leading cortical thickness measurements illustrating group-wise differences between our autistic and control subjects are mostly intra-regional variability with very few average thickness measurements. Table 3 summarizes our leading measurements-of-interest while providing bilateral effect size statistics across all age groups to help elucidate potential physiological characteristics of autism. In order to support a thorough analysis, all available FreeSurfer brain atlases were included in this study, resulting in Tables 2,3 reporting overlapping regions of interest including measurements across a region's cortex as well as localized gyral, sulcal, and lobular measurements when available.

**Table 3:** Summary of measurements with effect sizes potentially associated with known autistic characteristics.

**Autistic characteristics**	**Potentially associated measurements**	**Brain regions affected**	**Ages 0–5 L&R: d**	**Ages 5–10 L&R: d**	**Ages 10–15L&R: d**	**Ages 15–20L&R: d**
Facial & Visual processing	Inferior parietal cortex *SD*	Parietal	L (*d = –*0.47) R (*d = –*0.50)	L (*d =* 0.37) R (*d =* 0.34)	L (*d =* 0.51) R (*d =* 0.43)	L (*d =* 0.78) R (*d =* 0.71)
	Brodmann's area 1 *SD*	Parietal	L (*d = –*0.35) R (*d = –*0.35)	L (*d =* 0.00) R (*d =* −0.01)	L (*d =* 0.20) R (*d =* 0.14)	L (*d =* 0.57) R (*d =* 0.76)
	Intraparietal sulcus *SD*	Parietal	L (*d = –*0.54) R (*d = –*0.51)	L (*d =* 0.03) R (*d =* 0.15)	L (*d =* 0.44) R (*d =* 0.39)	L (*d =* 0.64) R (*d =* 0.77)
	Precuneus *SD*	Parietal	L (*d = –*0.57) R (*d = –*0.56)	L (*d =* 0.11) R (*d =* 0.17)	L (*d =* 0.29) R (*d =* 0.47)	L (*d =* 0.69) R (*d =* 0.72)
	Brodmann's area 2 *SD*	Parietal	L (*d = –*0.48) R (*d = –*0.42)	L (*d =* 0.13) R (*d =* 0.10)	L (*d =* 0.21) R (*d =* 0.29)	L (*d =* 0.51) R (*d =* 0.67)
	Inferior temporal cortex *SD*	Temporal	L (*d = –*0.41) R (*d = –*0.37)	L (*d =* 0.42) R (*d =* 0.35)	L (*d =* 0.49) R (*d =* 0.31)	L (*d =* 0.85) R (*d =* 0.72)
	Fusiform cortex *SD*	Temporal	L (*d = –*0.35) R (*d = –*0.25)	L (*d =* 0.69) R (*d =* 0.52)	L (*d =* 0.63) R (*d =* 0.49)	L (*d =* 0.62) R (*d =* 0.64)
	Superior temporal sulcus *SD*	Temporal	L (*d = –*0.52) R (*d = –*0.49)	L (*d =* 0.47) R (*d =* 0.43)	L (*d =* 0.63) R (*d =* 0.43)	L (*d =* 0.85) R (*d =* 0.76)
	Middle temporal visual area *SD*	Temporal	L (*d = –*0.43) R (*d = –*0.50)	L (*d =* 0.53) R (*d =* 0.55)	L (*d =* 0.60) R (*d =* 0.57)	L (*d =* 0.83) R (*d =* 0.73)
	Middle occipital gyrus *SD*	Occipital	L (*d = –*0.40) R (*d = –*0.35)	L (*d =* 0.54) R (*d =* 0.44)	L (*d =* 0.50) R (*d =* 0.42)	L (*d =* 0.90) R (*d =* 0.72)
	Brodmann's Area 18 mean	Occipital	L (*d = –*0.25) R (*d = –*0.24)	L (*d =* 0.33) R (*d =* 0.32)	L (*d =* 0.77) R (*d =* 0.67)	L (*d =* 0.71) R (*d =* 0.82)
	Brodmann's Area 17 mean	Occipital	L (*d = –*0.37) R (*d = –*0.29)	L (*d =* 0.50) R (*d =* 0.30)	L (*d =* 0.71) R (*d =* 0.61)	L (*d =* 0.60) R (*d =* 0.48)
	Lingual cortex *SD*	Occipital	L (*d = –*0.53) R (*d = –*0.40)	L (*d =* 0.19) R (*d =* 0.12)	L (*d =* 0.26) R (*d =* 0.13)	L (*d =* 0.71) R (*d =* 0.32)
	Cuneus gyrus mean	Occipital	L (*d = –*0.21) R (*d = –*0.24)	L (*d =* 0.49) R (*d =* 0.36)	L (*d =* 0.75) R (*d =* 0.60)	L (*d =* 0.62) R (*d =* 0.54)
Speech & Language processing	Triangular part of the inferior frontal gyrus *SD*	Frontal	L (*d = –*0.35) R (*d = –*0.31)	L (*d =* 0.17) R (*d =* 0.17)	L (*d =* 0.19) R (*d =* 0.19)	L (*d =* 0.68) R (*d =* 0.78)
	Pars triangularis *SD*	Frontal	L (*d = –*0.36) R (*d = –*0.33)	L (*d =* 0.09) R (*d =* 0.11)	L (*d =* 0.08) R (*d =* 0.26)	L (*d =* 0.58) R (*d =* 0.76)
	Brodmann's Area 45 *SD*	Frontal	L (*d = –*0.36) R (*d = –*0.26)	L (*d =* 0.18) R (*d =* 0.21)	L (*d =* 0.14) R (*d =* 0.23)	L (*d =* 0.55) R (*d =* 0.71)
	Supramarginal cortex *SD*	Parietal	L (*d = –*0.41) R (*d = –*0.53)	L (*d =* −0.06) R (*d =* 0.07)	L (*d =* 0.32) R (*d =* 0.24)	L (*d =* 0.73) R (*d =* 0.53)
	Superior temporal gyrus *SD*	Temporal	L (*d = –*0.29) R (*d = –*0.34)	L (*d =* 0.33) R (*d =* 0.45)	L (*d =* 0.54) R (*d =* 0.49)	L (*d =* 0.83) R (*d =* 0.91)
	Middle temporal gyrus *SD*	Temporal	L (*d = –*0.20) R (*d = –*0.32)	L (*d = –*0.26) R (*d =* 0.33)	L (*d =* 0.35) R (*d =* 0.56)	L (*d =* 0.69) R (*d =* 0.74)
	Lingual cortex *SD*	Occipital	L (*d = –*0.53) R (*d = –*0.40)	L (*d =* 0.19) R (*d =* 0.12)	L (*d =* 0.26) R (*d =* 0.13)	L (*d =* 0.71) R (*d =* 0.32)
Movement and Motor control	Precentral cortex *SD*	Frontal	L (*d = –*0.34) R (*d = –*0.30)	L (*d =* 0.19) R (*d =* 0.17)	L (*d =* 0.57) R (*d =* 0.11)	L (*d =* 0.70) R (*d =* 0.57)
	Brodmann's Area 6	Frontal	L (*d = –*0.37) R (*d = –*0.40)	L (*d =* 0.19) R (*d =* 0.12)	L (*d =* 0.44) R (*d =* 0.38)	L (*d =* 0.78) R (*d =* 0.70)
	Paracentral lobule and sulcus *SD*	Frontal	L (*d = –*0.44) R (*d = –*0.30)	L (*d =* 0.05) R (*d =* 0.10)	L (*d =* 0.17) R (*d =* 0.18)	L (*d =* 0.41) R (*d =* 0.66)
	Supramarginal cortex *SD*	Parietal	L (*d = –*0.41) R (*d = –*0.53)	L (*d = –*0.06) R (*d =* 0.07)	L (*d =* 0.32) R (*d =* 0.24)	L (*d =* 0.73) R (*d =* 0.53)
	Superior parietal *SD*	Parietal	L (*d = –*0.57) R (*d = –*0.51)	L (*d =* 0.12) R (*d =* 0.16)	L (*d =* 0.42) R (*d =* 0.39)	L (*d =* 0.74) R (*d =* 0.90)
	Intraparietal sulcus *SD*	Parietal	L (*d = –*0.54) R (*d = –*0.51)	L (*d =* 0.03) R (*d =* 0.15)	L (*d =* 0.44) R (*d =* 0.39)	L (*d =* 0.64) R (*d =* 0.77)
	Precuneus *SD*	Parietal	L (*d = –*0.57) R (*d = –*0.56)	L (*d =* 0.11) R (*d =* 0.17)	L (*d =* 0.29) R (*d =* 0.47)	L (*d =* 0.69) R (*d =* 0.72)
	Superior temporal gyrus *SD*	Temporal	L (*d = –*0.29) R (*d = –*0.34)	L (*d =* 0.33) R (*d =* 0.45)	L (*d =* 0.54) R (*d =* 0.49)	L (*d =* 0.83) R (*d =* 0.91)
	Lateral occipital cortex *SD*	Occipital	L (*d = –*0.48) R (*d = –*0.42)	L (*d =* 0.42) R (*d =* 0.27)	L (*d =* 0.38) R (*d =* 0.38)	L (*d =* 0.78) R (*d =* 0.55)
	Lateral occipital cortex mean	Occipital	L (*d =* 0.12) R (*d =* 0.01)	L (*d =* 0.01) R (*d =* 0.17)	L (*d =* 0.44) R (*d =* 0.56)	L (*d =* 0.69) R (*d =* 0.70)
	Brodmann's Area 18 mean	Occipital	L (*d = –*0.25) R (*d = –*0.24)	L (*d =* 0.33) R (*d =* 0.32)	L (*d =* 0.77) R (*d =* 0.67)	L (*d =* 0.71) R (*d =* 0.82)
Empathy deficits & Emotional processing	Lateral orbitofrontal cortex mean	Frontal	L (*d = –*0.23) R (*d = –*0.01)	L (*d =* 0.33) R (*d =* 0.29)	L (*d =* 0.60) R (*d =* 0.71)	L (*d =* 0.56) R (*d =* 0.52)
	Supramarginal cortex *SD*	Parietal	L (*d = –*0.41) R (*d = –*0.53)	L (*d =* −0.06) R (*d =* 0.07)	L (*d =* 0.32) R (*d =* 0.24)	L (*d =* 0.73) R (*d =* 0.53)
	Inferior parietal cortex *SD*	Parietal	L (*d = –*0.47) R (*d = –*0.50)	L (*d =* 0.37) R (*d =* 0.34)	L (*d =* 0.51) R (*d =* 0.43)	L (*d =* 0.78) R (*d =* 0.71)
	Precuneus *SD*	Parietal	L (*d = –*0.57) R (*d = –*0.56)	L (*d =* 0.11) R (*d =* 0.17)	L (*d =* 0.29) R (*d =* 0.47)	L (*d =* 0.69) R (*d =* 0.72)
	Superior temporal sulcus *SD*	Temporal	L (*d = –*0.52) R (*d = –*0.49)	L (*d =* 0.47) R (*d =* 0.43)	L (*d =* 0.63) R (*d =* 0.43)	L (*d =* 0.85) R (*d =* 0.76)

*R, right; L, left; SD, standard deviation; d, Cohen's d statistic*.

We have performed a preliminary validation with the ABIDE dataset (Di Martino et al., 2014). We have elected to perform a preliminary validation analysis assessing the leading five feature measurements identified in our findings (first five rows of Table 2) against the ABIDE imaging center with the most participants aged 15–20 at imaging (the USM-ABIDE data) as this age range exhibited the largest group-wise differences in our study. Results confirm four out of our leading five cortical thickness variability measurements with reduced diagnostic potential relative to our BCH data: left superior temporal (BCH: AUC = 0.73, ABIDE: AUC = 0.64), right superior parietal (BCH: AUC = 0.74, ABIDE: AUC = 0.62), right Brodmann's area 6 (BCH: AUC = 0.71, ABIDE: AUC = 0.58), right superior temporal sulcus (BCH: AUC = 0.68, ABIDE: AUC = 0.61), and the left middle occipital gyrus (BCH: 0.74, ABIDE: 0.50). This confirmed our findings in four of our five leading measurements (all but the middle occipital gyrus), albeit with reduced separation between the control subjects and autistic groups in the ABIDE dataset. Reduced separation in the ABIDE dataset relative to BCH data may be caused by differences in distributions of autistic severity in each group with the routine clinical BCH data likely to exhibit increased proportions of unhealthy autistic children (those with comorbidities) which may also be correlated with more severe manifestations of autism. In contrast, the ABIDE dataset's autistic population's intelligence quotients (IQ) are similar to their control subject counterparts (Di Martino et al., 2014), implying that the dataset might disproportionately represent children with high functioning autism. While we were unable to confirm abnormal cortical thickness variability in the middle occipital gyrus in the ABIDE dataset, we did observe abnormal average cortical thickness in the left middle occipital gyrus (AUC = 0.64) and abnormal cortical thickness variability in the inferior occipital gyrus (AUC = 0.61) in this preliminary ABIDE validation.

## Discussion

We performed a large-scale cortical thickness analysis of structural MRI examinations of the brain in autistic and neurotypical individuals and demonstrated group-wise differences in cortical thickness variability as well as average values localized to select regions across the brain. Many brain regions showed differences in intra-regional variability of the cortical thickness, which was reported for the first time in this study. Atypical developmental patterns have been detected as early as 0 years old in individuals who would later be diagnosed with autism. We have confirmed our results using a publicly open database. Reduced cortical thickness variability was observed in the early years followed by abnormally increased variability in later years in autism.

### Potential Association Between Our Findings and Known Symptoms of Autism

The majority of the leading measurements of interest identified in this study have potential to be associated with known outward symptoms and characteristics (endophenotypes) of autism (Table 3), including disorders of visual and facial processing (Behrmann et al., 2006), empathy and emotional processing (Jones et al., 2010), speech and language processing (Kellerman et al., 2005; Wan and Schlaug, 2010), as well as movement and motor control (Dziuk et al., 2007).

We found brain regions potentially associated with disorders of visual facial processing including the left inferior temporal region (Haxby et al., 2000) (Figure 3), the bilateral inferior parietal region, which is involved in the perception of emotions in facial stimuli (Radua et al., 2010), the left superior temporal sulcus, which has been claimed to be involved in the perception of where others are directing their gaze, and is thought to be important in determining where others' emotions are being directed (Campbell et al., 1990), the right fusiform region, which has been observed to influence the amygdala's response to emotional faces (Stephanou et al., 2016) and activation therein has been observed in autistic participants viewing faces (Hadjikhani et al., 2004), the middle occipital gyrus, which is involved in visual processing, Brodmann's areas 1 (image texture processing), 2 (object size and shape processing), and areas 17 and 18, which are involved in primary visual processing and for which abnormal activation has been observed among the autistic (Soulieres et al., 2009; Clery et al., 2013), the intraparietal sulcus, which is involved in visual attention, the precuneus which is involved in visuo-spatial imagery (Cavanna and Trimble, 2006), and finally, the lingual region, which appears to provide input to the ventral face area (McCarthy et al., 1999).

Identified brain regions potentially associated with disorders of speech and language include the bilateral superior temporal region (Figure 3) and Heschl's gyrus, which contain Brodmann's areas 41, 42, 22, representing the primary and part of the association auditory cortex (Bigler et al., 2007), pars triangularis (bilaterally) (Brodmann's area 45, triangular part of the inferior frontal gyrus), corresponding to Broca's language area in the left frontal lobe, the supramarginal gyrus, which may be associated with language function as lesions therein may cause receptive aphasia (Gazzaniga et al., 2009), the middle temporal gyrus, which has been identified as a critical node in the brain's language network (Acheson and Hagoort, 2013) and finally, the lingual and fusiform regions, which have been shown to be involved in language tasks (Mechelli et al., 2000).

Measurements demonstrating group-wise differences were also found in brain regions potentially associated with movement and motor control disorders including: the bilateral supramarginal gyrus, which is involved in the perception of space and limb locations, the left lateral occipital region and the bilateral inferior temporal gyrus (Brodmann areas 18, 19, 37), which are involved in visual object recognition (Logothetis and Sheinberg, 1996), the precentral gyrus, which is the site of the primary motor cortex (Brodmann's area 4), the superior parietal region, which is thought to be involved with spatial orientation, Brodmann's area 6, which contains the premotor cortex and in which abnormal activation has been observed among autistic participants relative to control subjects (Mostofsky et al., 2009; Barbeau et al., 2015), the intraparietal sulcus, which incorporates visual control with motor movements, the precuneus, which is involved in attention to motor targets (Cavanna and Trimble, 2006), the paracentral sulcus and lobule, which corresponds to the supplementary motor area, and finally, the superior temporal cortex (Figure 3), whose right hemisphere mediates spatial awareness and exploration (Karnath, 2001).

Regions potentially linked to empathy deficits and disorders of emotional processing include the bilateral orbitofrontal region, which forms the basis for an existing test for autism (Stone et al., 1998), the superior temporal gyrus (Figure 3) and the bilateral inferior parietal region, which is involved in the perception of emotions in facial stimuli (Radua et al., 2010), the bilateral supramarginal region, which is thought to be associated with empathy (Silani, 2013), and finally, the precuneus, which has been shown to activate when a participant decides whether to act out of empathy or forgiveness (Farrow et al., 2001).

Additionally, the bilateral middle frontal region, which is thought to be involved in episodic memory retrieval (Rajah et al., 2011), also exhibits abnormalities among autistic participants, which may have a pervasive impact on the development of other brain regions if such development is reliant on recalling past stimuli.

Variability in cortical thickness may be indicative of underlying structural abnormalities prevalent among individuals with autism. These findings potentially implicate abnormal gray matter development among autistic participants. Given that group-wise comparisons demonstrate abnormally reduced cortical thickness variability in the early years followed by abnormally increased variability in later years in autism, it is possible that autism or autism-susceptible individuals tend to have late-onset cortical development followed by a rapid, “excessive maturation” potentially caused by genetic and/or environmental effects in a participant's teenaged years. It is also possible that genes linked to pubertal development are associated with the inversion of this effect in a participant's teenaged years.

### Strengths of This Study

The main strength of this research is that it is the largest single center study of its type in terms of the number of exams and includes a wide range of developmental ages among the study's participants. This retrospective analysis included a cohort of very young participants who received imaging prior to their diagnosis of autism, a population difficult to include in a traditional prospective study design, which typically requires recruitment of participants based on a pre-existing diagnosis. This work also involved incorporating intra-regional variability of cortical thickness measurements (Fischl, 2012), making this study more thorough than typical approaches that focus only on average cortical thickness measurements (Jiao et al., 2010; Zielinski et al., 2014). Results of our study indicated that for many sub-regions of the brain, the most discriminating measurements at multiple age groups was the variability (standard deviation) of the cortical thickness, a measurement that does not appear to have been considered in studies published in the literature.

Our dataset includes many examinations of participants aged 0–2.5 years, providing data on early stages of autism's development that is minimal in the scientific literature. Many of our youngest patients were imaged with MRI prior to their autism diagnosis. By including all samples available, we provide a thorough analysis of a clinical population, which is ideal for the assessment and development of diagnostic tests that ultimately would be applied to autistic participants who receive routine clinical imaging. Future generations of diagnostic technologies will be responsible for the correct identification of a variety of pathological conditions (autism included) from large pools of participants assessed with routine clinical imaging, making clinically imaged autistic participants an interesting population for further research despite this group not having been studied in-depth to date.

### Relationship With Existing Literature Findings

MRI data acquisition involves measurement noise. Additional noise can be introduced by FreeSurfer technology. Furthermore, there is a natural amount of variability in both the control subjects investigated, as well as in our autistic participants. These factors result in substantial measurement variability when employing MRI and FreeSurfer to assess autistic and control subjects. This variability may explain the inconsistencies reported in the many MRI-based autism FreeSurfer studies that have been published in the literature, which are based on relatively small sample sizes, from widely ranging age groups, none of which cover the entire age range from newborn to adult (Groen et al., 2010; Jiao et al., 2010; Schumann et al., 2010; Schaer et al., 2013, 2015; Wallace et al., 2013; Ecker et al., 2014; Zielinski et al., 2014; Richter et al., 2015; Yang et al., 2016). With few samples available, differences between autistic and control subjects may appear to exist when the observed effect could merely be a by-product of high levels of measurement variability. With high measurement variability, few samples and many measurements evaluated, some measurements will exhibit substantial group-wise differences by chance. Insufficient sample sizes in the presence of high levels of measurement variability can lead to erroneous findings. Measurement variability can also obscure real effects as non-statistically significant when sample sizes are very low. Despite these shortcomings, our study was able to confirm literature findings of lowered average cortical thickness in the left parahippocampal region (6–15 years, AUC = 0.57, *p* = 3.69e^−5^) and in the left frontal pole (6–15 years, AUC = 0.61, *p* = 7.84e^−8^) as well as increased average cortical thickness in the left caudal anterior cingulate (6–15 years, AUC = 0.58, *p* = 5.48e^−6^) and increased thickness in the left precuneus (6–15 years, AUC = 0.58, *p* = 4.10e^−5^) in agreement with literature findings (Jiao et al., 2010). We were also able to confirm increased average cortical thickness in the left pars opercularis (12–32 years, AUC = 0.62, *p* = 4.33e^−5^), left rostral middle frontal (12–32 years, AUC = 0.60, *p* = 3.93e^−6^), left frontal pole (12–32 years, AUC = 0.61, *p* = 5.01e^−7^), right paracentral (12–32 years, AUC = 0.55, *p* = 0.016), and the right lateral occipital region (12–32 years, AUC = 0.68, *p* = 3.11e^−18^) in agreement with literature findings (Zielinski et al., 2014). Our primary findings pertain to abnormal variability in regionally assessed cortical thickness in the developing autistic brain. When measurement variability is high, the number of samples required to have confidence in reported findings increases and provides motivation for conducting traditional average cortical thickness studies with large numbers of participants, in order to assist in producing literature findings that are consistent with one another.

### Limitations

A major limitation of this study is a lack of gold standard diagnoses for autism (ADI-R and ADOS evaluations were unavailable). This problem is caused by the retrospective nature of this study, for which it was not feasible to interview each participant and thus electronic patient medical records were relied upon. While indications of autism are typically entered into the electronic patient medical records by a Boston Children's Hospital physician, this does not guarantee that our dataset does not include participants whose autistic status was established by a community physician who is not an expert in diagnosing autism. Additionally, intelligence quotient (IQ) information was unavailable for the participants in this study. The retrospective nature of this study makes it impossible to account for all variables tracked and controlled for in prospective studies, which include detailed participant interviews, but it is hoped that this work will identify physiological effects of interest that will be thoroughly validated in carefully controlled prospective studies as part of future work. It is also hoped that this work can help bridge the gap between prospective studies and what can be achieved clinically. An additional limitation of our study was the need to procure control subjects that were inferred to be typically developing from a routine clinical population. This was accomplished by excluding participants with indications of a long list of neurological issues while requiring each participant's MRI examination to have been assessed as normal by a BCH neuroradiologist (Levman et al., 2017). This process yielded 993 examinations from participants deemed most likely to represent control subjects from a large pool of MRI examinations, in order to best approximate a control population from large-scale routine clinical imaging. It is expected that the rate of false negatives (seemingly normal participants who in fact have a neurological issue) might be higher than the rate exhibited in typical well-controlled prospective studies and so may add variability to our control measurements and may represent an additional source of error in our study.

An additional limitation of this study is that it was performed retrospectively on participants that received imaging for a wide variety of reasons. Among the control subjects, the leading reasons for the MRI examinations were headaches (60%), to rule out intracranial pathologies (13%), vomiting (11%), and night awakenings (10%). Among our autistic participants, the leading reasons for the MRI examinations were seizures (19%), to rule out intracranial pathologies (14%), and an abnormal EEG (9%). Since the population was drawn from routine clinical imaging, there is also a wide variety of comorbidities indicated in many of our participant's electronic medical records. The most common comorbidities in our control subjects are migraines (23%), headaches (19%), and abdominal pain (11%). The most common comorbidities in our autistic group are global developmental delay (26%), attention deficit hyperactivity disorder (16%), abdominal pain (14%), and epilepsy (13%). This study design was intended to provide a thorough analysis of a complete clinical population, providing a baseline of what to expect from other clinical populations and facilitating research into the next generation of diagnostic tests, which would be applied to populations akin to the one investigated in this study. Traditional MRI studies often involve imaging participants who are much healthier than is clinically realistic. The study design presented here allows for the assessment of what can be accomplished in a large-scale clinical context.

There is some variability in imaging parameters (spatial resolution, signal-to-noise ratio etc.) caused by variations in the pulse sequences employed; however, imaging was performed with a consistent set of 3 Tesla Siemens MRI scanners all installed at BCH in 2007. Ideally, this study would be performed on scans using a single MRI protocol; however, doing so would greatly reduce the number of samples available for inclusion in this analysis. Large sample sizes help to overcome potential bias associated with measurements that exhibit considerable variability. While limiting the analysis to a single imaging protocol would reduce potential bias caused by scan parameter variability, it would increase bias caused by sample size effects. Many measurements produced by FreeSurfer on our BCH dataset demonstrate that the discriminating power (between autistic and control subjects) of volumetric measurements (in mm^3^) is approximately identical to the discriminating power of the voxel counts in those same regions (this includes ventricular volumes/voxel counts and corpus callosum volumes/voxel counts). Since voxel counts vary greatly based on spatial resolution variations, we believe the effect on our results caused by varying spatial resolutions in our MR protocols to be modest. In addition, we compared our large sample size findings with findings from an independent analysis performed at a single center with a single pulse sequence (USM-ABIDE). Thus, our primary findings have been confirmed independently with data that does not suffer from the issues.

An additional limitation of this study is that the age distributions of available participants for the two groups in this experiment vary considerably (Figure 2), because of the availability of appropriate participants that met our inclusion criteria from a large clinical population. This inevitably resulted in imbalanced pools of participants for further analysis. Our experiment did not involve age- or gender-based participant matching between our autistic and control subjects. Instead, we have opted to perform our statistical analyses in a group-wise manner, varying the age range under consideration, and to plot our main findings on an age-dependent basis while differentiating between male and female participants in our scatter plots. This methodology was selected to avoid the reduced sample size that would arise from only including those autistic participants who have a control subject counterpart with the same gender and identical age. Additionally, this methodology was selected in order to avoid having our analysis be influenced by the extent of difference between matched pairs of individuals, for which a variety of factors beyond age and gender might influence how appropriate it was for the participants to have been paired (brain volume, sub-structure volume, co-morbidities, etc.). We also performed a multivariate regression analysis that controls for the effects of age, gender, intracranial volume and several comorbidities in order to confirm that these factors aren't the cause of our reported findings. Comparative assessment of males and females from our control subjects revealed no major gender differences in terms of either mean or the standard deviation of the cortical thickness measurements. A large gender-segregated analysis of 442 control subjects has also been performed (Koolschijn and Crone, 2013) which did not identify our primary findings as exhibiting gender differences.

A variety of alternatives to the stringent Bonferroni correction were considered as alternative statistical analyses to be relied upon in this study. As a large-scale review of real-world clinical data, we are presenting an analysis of a considerably different type than is common in the literature. Our dataset, while having standardization advantages over many clinical centers (Boston Children's Hospital installed a suite of 3T Skyra Siemens MRI scanners in 2007, while most clinical centers have a variety of different MRI scanners), we have standardization disadvantages relative to typical prospective studies (in which all T1 volumetric examinations are normally acquired with an identical MRI pulse sequence). This inevitably introduces additional variability/error in our measurements and when designing our analytic strategies for assessing our data, we felt it important to be particularly cautious when presenting an effect that appears to be associated with the presentation of autism clinically. This is why the most stringent accepted method was used, to reduce the false discovery error rate and thus limit the likelihood that our analysis reports findings that will not be confirmed in future studies. Type II errors were of far less concern to us, as this is akin to accidentally declaring no effect associated with autism when a real effect was present. Our analysis is most concerned with assessing the largest effects and we recognize that a heterogeneous clinical population is not likely to be the best method available for assessing the existence of small effect sizes, thus our reduced concern for type II errors, which in turn lead to our decision to employ the extra-stringent Bonferroni correction in this analysis. Additionally, it should be noted that this was not a paired analysis, but instead a group-wise analysis performed on a large-scale real-world clinical population. In order to analyze a complete set of clinical data, there are inevitably differences between the two populations in terms of gender and age distributions (note that there are about 4 males with autism for every female, whereas there is about 1 control subject male for every female). The issues associated with these imbalances were addressed by comparing our results with an independent dataset (i.e., through external validation) and by employing multivariate linear regression to perform secondary analyses that demonstrate that our findings are still statistically significant after controlling for the effects of gender and age.

An additional limitation of this study is that FreeSurfer is not optimized for the youngest participants in our analysis. As such, the rate at which FreeSurfer fails to extract measurements from clinical MRI examinations increases substantially for participants aged 0–8 months and the reliability of the results successfully produced by FreeSurfer on participants from this age range is uncertain. FreeSurfer's reliability was assessed as reasonable for participants 8-months-old and later (considering this is beyond the age range for which the technology was validated), at which point myelination contrast patterns have inverted so as to match the general pattern exhibited through the rest of life (with gray contrast located on the brain's periphery and white contrast occupying central regions). Research aimed at overcoming the problem of FreeSurfer's applicability and reliability in very young populations is ongoing (de Macedo Rodrigues et al., 2015; Zollei et al., 2017) and any developments in this venue will be incorporated into future work.

### Future Work

In addition to incorporating infant FreeSurfer atlases, we will also extend this analysis to tractography, functional MRI (fMRI) and multivariate machine learning as well as to perform a detailed and thorough validation with the ABIDE dataset. Additional future work will involve correlating our dataset with detailed clinical information not available in the electronic patient medical records. This large-scale task may allow us to assess the potential association between MRI measurements and symptom severity, participant outcomes etc. Future work will also look at comparing the autistic group with groups at high risk for autism and groups that are clinically similar to autism in presentation in order to extend this work's diagnostic assessments to differential diagnosis.

Our results indicate that automatically extracted measurements can be used to predict the pathological status of a participant whose brain has been imaged with MRI; however, future work is needed to optimize the performance of such a diagnostic test. We hope that these research avenues will assist toward better understanding autism as well as improved characterization, diagnosis and classification of the disorder into subtypes.

## Author Contributions

PM, NS, and AL were responsible for data acquisition and analysis. BE and SR were responsible for the ABIDE validation. AG provided detailed feedback on study findings and their possible relation to brain function as well as manuscript editing. JL and ET designed the study and supervised PM, NS, and AL jointly. JL supervised BE and SR.

### Conflict of Interest Statement

The authors declare that the research was conducted in the absence of any commercial or financial relationships that could be construed as a potential conflict of interest.
